# Epidemic Cholera

**Published:** 1892-10

**Authors:** 


### Editorial.

### EPIDEMIC CHOLERA.

At one time there seemed no doubt of cholera gaining a foothold in the United States, for the most rigid quarantine measures that any American port has ever exercised have heretofore proved ineffectual, and seven deaths did occurr in New York city. These deaths occurred in different sections of the city, and how the persons dying became infected with the disease we know not. We only know that in some manner the agent carrying the specific cause of the disease passed quarantine, and that quarantine regulations proved ineffectual in preventing its advent, although they may have prevented an epidemic at the present time by arresting a large number of persons who would have proved centers of infection. Whether quarantine regulations will prevent the occurrence of an epidemic next spring is the question. Cholera may be introduced into this country in three ways: first, by passengers who are apparently in good health, but who in reality contain cholera germs; second, by provisions; and third, by freight, such as hides, rags, etc.

Quarantine has no doubt prevented the introduction of the disease by the landing of infected passengers, for we can trace no case as yet to any one who has passed through quarantine; but can quarantine prevent the introduction of cholera germs in provisions, and ship-freight brought to this country? A vessel without a single case aboard when it lands, and with no history of disease on its passage, yet may have the germ of the disease in its freight, and when this freight is handled in landing, and shipped to its final destination (which may be some inland town instead of a seacoast city,) then the germs which have lain dormant during the voyage, because the cargo has not been handled by the sailors, may find the proper soil for its culture and prove a center of infection. It is impossible to disinfect large cargoes of freight so thoroughly that all germs of disease will be destroyed.

If quarantine is kept up for eight or ten months, and all importation is prohibited, then it may be possible that the cholera can be kept away, but as soon as strict quarantine is relaxed and importation is allowed, the germs of cholera will surely be introduced.

The seven sporadic cases of cholera in New York city have been caused, no doubt, by poison which passed into this country in this manner, and probably before the quarantine regulations were vigorously enforced. We trust that no more cases will occur, but we have the dread of an epidemic in the future, and we will from now on have to guard against anything that may prove favorable to its origin. Sporadic cases can occur in clean, healthy neighborhoods, but bad local conditions are necessary for epidemics. Water is the chief means by which the disease is spread. One infected well or spring may cause hundreds of cases, and the water supply of Atlanta, infected by drainage into the reservoir from its water shed of the cholearic discharges of one person would cause an epidemic that would decimate the city. Too much attention cannot be paid to the water supply, cleaning of all the streets, alleys, backyards, sewers, and low places, for the only thing that will bring absolute immunity from epidemic cholera will be the purest water and the strictest cleanliness.

A most important step to take in preventing epidemics, especially Asiatic cholera, is to dispose properly of the dead. The few cases that have died in New York have been buried in hermetically sealed metallic caskets. This, in my opinion, is only prolonging the evil. I have seen several of these caskets exhumed, and all of them had been burst by an expansion of the gases due to decomposition, thereby allowing an escape of the fluid contents. The metallic casket is likely to do more harm than good by giving a fancied security, when if a wood casket had been used it would not have been. It is possible that graveyard drainage may infect the water supply. In fact, Dr. John G. Lee, of Philadelphia, says, "Burial in the soil within and near our large cities pollutes the earth, the air and water; the earth has no time to destroy the germs of contagious diseases, and the air and water are, when infected, active agents in their propagation."

Three cemeteries of Philadelphia, containing 80,000 graves, are so situated as to render it possible for them to drain into the Schuylkill river which furnishes drinking water to more than one million of people, therefore, it is possible that in our cemeteries we may have a continuous source of infection. We should find other means than inhumation for the disposal of the dead, and if not in death from ordinary diseases, certainly in death from contagious and infectious diseases.

In cremation, we have the only real scientific method, and the only method that removes forever all fear of further danger from any body dying of a contagious or infectious disease. The dead from cholera in Buenos Ayres, during their last epidemic, were cremated, and cremation was made compulsory during the epidemic, which was a small one, nearly a thousand bodies being cremated.

It is customary in some cities to have their pauper dead, and those dying during epidemics, buried in pits about eight feet square and twelve deep, putting ten or twelve bodies in one pit, and allowing the pit to remain open except for a few inches of dirt thrown over the upper layer of coffins, until the required number of bodies have been placed therein. This practice cannot be too severely censured, for it must prove a prolific source of disease, whether the bodies are of infectious diseases or not. Only last fall, I saw five cases of typhoid fever in one house at the same time, and no cause could be found unless it was the well-water which ran from the direction of a fat graveyard on a slightly higher elevation and only eighty yards off. This stream of water ran along a lime-stone formation which lay under the graveyard and slanted in the direction of the well, the soil above being a light sandy loam and very porous.

To prevent an epidemic of cholera, all bodies dying of the disease should be at once cremated, and if this is done while we have only a few sporadic cases, and these sporadic cases are isolated as soon as the disease is recognized, then we may, by this vigorous means, stamp out the poison before it has gained strength enough to rise up and cause a mighty epidemic.

